# The analysis of APOL1 genetic variation and haplotype diversity provided by 1000 Genomes project

**DOI:** 10.1186/s12882-017-0675-6

**Published:** 2017-08-11

**Authors:** Ting Peng, Li Wang, Guisen Li

**Affiliations:** Renal Division and Institute of Nephrology, Sichuan Academy of Medical Sciences and Sichuan Provincial People’s Hospital, School of Medicine, University of Electronic Science and Technology of China, No. 32, West 2nd Duan, 1st Circle Road, Qingyang District, Chengdu, Sichuan People’s Republic of China 610072

**Keywords:** Apolipoprotein L1, Haplotype, Single nucleotide polymorphisms, Genetic diversity, 1000 Genomes Project

## Abstract

**Background:**

The APOL1 gene variants has been shown to be associated with an increased risk of multiple kinds of diseases, particularly in African Americans, but not in Caucasians and Asians. In this study, we explored the single nucleotide polymorphism (SNP) and haplotype diversity of APOL1 gene in different races provided by 1000 Genomes project.

**Methods:**

Variants of APOL1 gene in 1000 Genome Project were obtained and SNPs located in the regulatory region or coding region were selected for genetic variation analysis. Total 2504 individuals from 26 populations were classified as four groups that included Africa, Europe, Asia and Admixed populations. Tag SNPs were selected to evaluate the haplotype diversities in the four populations by HaploStats software.

**Results:**

APOL1 gene was surrounded by some of the most polymorphic genes in the human genome, variation of APOL1 gene was common, with up to 613 SNP (1000 Genome Project reported) and 99 of them (16.2%) with MAF ≥ 1%. There were 79 SNPs in the URR and 92 SNPs in 3’UTR. Total 12 SNPs in URR and 24 SNPs in 3’UTR were considered as common variants with MAF ≥ 1%. It is worth noting that URR-1 was presents lower frequencies in European populations, while other three haplotypes taken an opposite pattern; 3’UTR presents several high-frequency variation sites in a short segment, and the differences of its haplotypes among different population were significant (*P* < 0.01), UTR-1 and UTR-5 presented much higher frequency in African population, while UTR-2, UTR-3 and UTR-4 were much lower. APOL1 coding region showed that two SNP of G1 with higher frequency are actually pull down the haplotype H-1 frequency when considering all populations pooled together, and the diversity among the four populations be widen by the G1 two mutation (*P*_1_ = 3.33E-4 vs *P*_2_ = 3.61E-30).

**Conclusions:**

The distributions of APOL1 gene variants and haplotypes were significantly different among the different populations, in either regulatory or coding regions. It could provide clues for the future genetic study of APOL1 related diseases.

**Electronic supplementary material:**

The online version of this article (doi:10.1186/s12882-017-0675-6) contains supplementary material, which is available to authorized users.

## Background

The apolipoprotein L1 protein is a 43 kDa protein belonged to the lipocalin family and has 4 splice variants encoding 3 different isoforms: variants 1 and 3 encode the same apolipoprotein L1 isoform a, variant 2 and 4 encode isoform b and c precursor, respectively. APOL1 plays an important role in the trypanosomal lysis [1–4], autophagic cell death [5, 6], lipid metabolism [7–9], as well as vascular and other biological activities. The main features that distinguish APOL1 from the other members of APOL genes family are, (a) APOL1 gene is in the opposite orientation to the other three (APOL2, APOL3, APOL4); (b) it encodes the only secreted protein in the family; (c) APOL1 plays an important role in trypanosomal lysis; (d) it also acts as a risk gene for many kinds of kidney diseases.

Apolipoprotein L1 (APOL1) belongs to the family of Apolipoprotein L genes, located at Chromosome 22q13. APOL1 gene (Gene ID: 8542) encompasses a region of 14,461 nucleotides and presents seven exons and six introns, and encodes mRNA of 3039 nucleotides. Considering the full-length mRNA (NM_145343.2), 1245 nucleotides represent coding segment (CDS) encoding 414 amino acids, 274 nucleotides present in 5′ untranslated region (5’UTR) segment, and 1520 nucleotides represent the 3′ untranslated region (3’UTR) segment. The APOL1 CDS is composed of joining segments of six exons. The APOL1 protein has signal peptide (SP), pore forming domain (PFD), membrane-addressing domain (MAD) and SRA-interacting domain [4, 10]. Part of the signal peptide is encoded by exons 2, exon 3 and exon 4. Exon 6 encodes the PFD. The exon 7 with 2381 nucleotides is 3.7 times as much as the sum of other six exon nucleotides and accounts for 78% of the whole mRNA sequence. Therefore, the exon 7 encodes three function domains that include the part of PFD, full length MAD and SRA-interacting domain (Additional file 1: Table S1).

The influences of APOL1 to innate immunity and susceptibility to kidney disease [11–13] have been extensively studied since its discovery by Duchateau, et al. in 2001 [14]. Numerous studies have revealed that the functional mutations of APOL1 associated with African narcolepsy [1, 15], atherosclerosis [16, 17], schizophrenia [18, 19], cancer [20] and other diseases. The two variants (G1: rs73885319 A > G, and rs60910145 T > G; G2: rs71785313 TTATAA/−) of APOL1 has been shown to be associated with an increased susceptibility of multiple kinds of kidney diseases, particularly in African Americans. These kidney diseases included focal segmental glomerulosclerosis (FSGS), hypertensive nephropathy (HTN), human immunodeficiency virus associated nephropathy (HIVAN), etc. [21–25]. The two risk variants could also increase the severity of these kidney diseases and the risk of progress to end-stage renal disease (ESRD) [21–25]. The previous reports showed that APOL1 variants could increase the risk of CKD and ESRD in patients with HIV infection [24, 26, 27]. But unfortunately, the associations were not well validated in Caucasian and Asian populations.

In our previous study, we haven’t found the two risk variants (G1 or G2) of APOL1 in Chinese CKD patients [28]. It suggested that there was a significant difference in the variation of APOL1 among different races, and there might be other variations in the APOL1 gene, rather than G1 or G2, associated with the kidney diseases in Caucasian or Asian population. We need to explore the differences in APOL1 variability among different races, in order to provide more information on future genetic studies on APOL1-related kidney disease.

The 1000 Genomes Project is a large survey aiming to sequence the entire genome of thousands of individuals in several populations around the world [29, 30]. It can help researchers to investigate the relationship between genotype and phenotype and understand the genetic contribution to disease [31]. In this study, we explored the characteristics of APOL1 gene variation in different races based on the 1000 Genomes Project database. It would be helpful for the future study which concerned the associations between APOL1 gene variations and kidney diseases.

## Methods

### Accession and filtration of SNPs of APOL1 gene from 1000 genomes project

We analyzed all the SNPs data of APOL1 derived from 1000 Genomes Phase 3 Pipeline, included 2504 individuals from 26 human populations as described in NCBI Variation Glossary (http://www.ncbi.nlm.nih.gov/variation/docs/glossary). Through the comprehensive consideration, we classified those populations as four race included Asia, Africa, Admixed and Europe (Additional file 1: Table S2) [30, 32, 33]. First, we downloaded the VCF files containing the all SNP for APOL1 gene region (between positions 36,649,117 and 36,663,577 at chromosome 22) directly from the 1000 Genomes server (ftp.1000 genomes.ebi.ac.uk//vol1/ftp/). A total of 2028 SNPs across APOL1 was identified using the dbSNP database (http://www.ncbi.nlm.nih.gov/SNP/), while the number of APOL1 SNPs officially released by 1000 Genomes Project is 612. The two sequences defined by NCBI (DNA: NG_023228.1, and mRNA: NM_145343.2) were used as references in the study.

Generally, a SNP is considered as a true polymorphic site if its minor allele frequency (MAF) presents at least 1%. In this matter, we get 100 SNPs filter by MAF ≥ 1% in APOL1 gene region. As the conventional nomenclature, we consider the first base in APOL1 gene sequence as nucleotide +1, and classify the functional effect of each SNP as intronic, coding synonymous mutations, coding missense mutations, 5′ untranslated region (UTR) and 3’UTR (according the NCBI SNP annotation and Variation Viewer) (Additional file 1: Table S3). Only the SNPs located in the regulatory region or exon region were included for further analysis. Considering the annotation of APOL1 in NCBI (NM_145343.2), the first base of APOL1 initial codon ATG located at the nucleotide 893. The previous study indicated that there were many regulatory elements in the APOL1 gene upstream [14]. In this scenario, we defined nucleotides between −1200 and +892 (included APOL1 gene upstream and 5′ untranslated region) as CDS upstream regulatory region (URR) to include all regulatory elements. The 3’UTR covered 1497 nucleotides from nucleotide 12,964 to 14,461.

Then we checked the 100 SNPs reference allele, alter allele and MAF in NCBI and Ensemble database compared with 1000 Genomes Browser to ensure the integrity and correctness of the information. For example, rs74904227, rs136162, rs136163, rs80424, rs136174 and rs189436505, the six SNP sites were the triallelic alleles according NCBI and Ensemble, which be properly corrected as per 1000 Genome data (Although a triallelic SNP is described at NCBI, we did not find the third allele in 1000 Genome data). In the Allele2 column of Additional file 1: Table S3, the underline bases indicate the standard alter allele in 1000 Genome data. However, NCBI, Ensemble and 1000 Genomes data indicated that rs136162 was trialleic, so this polymorphism site was discarded to keep the consistency of the data format.

### Tag SNPs selection and statistical analysis

Haploview 4.2 software [34] was used to analyze linkage disequilibrium (LD) among the 99 variation sites. It showed that the LD was stronger within a block, and the number of major haplotypes was limited [35]. Hardy–Weinberg equilibrium *p* value (HWp) was calculated for each SNP by Haploview 4.2 software. We used a set of tagging SNPs selected from LD data and tagger option of Haploview to predict values of remaining SNPs in the dataset [36], combined the block-based and block-free approach [37] to improve the representativeness and accuracy of tag SNP, and assess the accuracy of those tag SNP by evaluating the value of r^2^ and D’ in Haploview analysis. We chose Tag SNP for APOL1 URR, coding region and 3’UTR region respectively, which presented relative higher MAF and meet the HW equilibrium tested by Haploview software. Finally, the selected tag SNPs were used to evaluate the haplotypes in the four populations by HaploStat software (version 1.7.7) [38]. Chi-square statistical method was used to test the differences of each haplotype among four populations.

## Results

### APOL1 variability as described in the 1000 genomes project

**Fig. 1:**
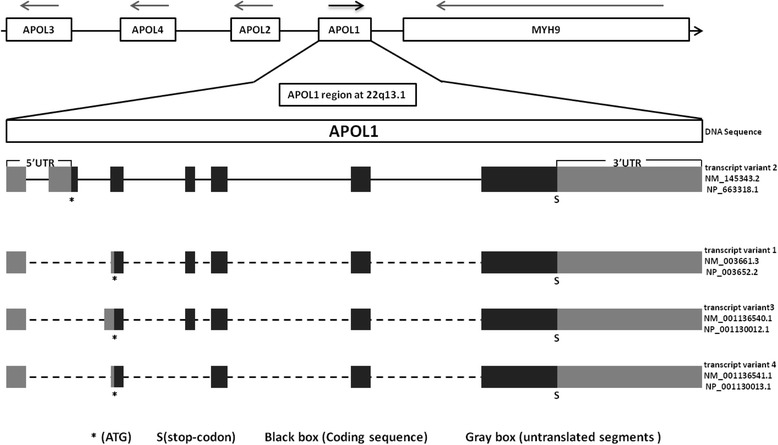
APOL1 gene structure and transcripts (NCBI notations)

APOL1 gene was surrounded by some of the most polymorphic genes in the human genome (Fig. 1) such as APOL2 (13,117 bp upstream), APOL3 (86,894 bp upstream), and APOL4 (48,238 bp upstream), and other non-APOL family loci such as MYH9 (13,746 bp downstream). APOL1 gene had four LD blocks (Additional file 1: Figure S1) and 43 variants conformed to HWp > 0.05. A total of 12 polymorphism loci distribution (**bold SNP**) in APOL1 whole segment were selected as tag SNP (Additional file 1: Figure S2).

**Fig. 2:**
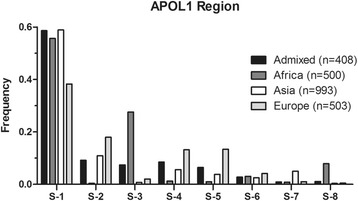
Frequency distribution of APOL1 whole segment haplotypes in different populations

There were 8 haplotypes with a global frequency higher than 1% (Table 1). The frequency of each haplotype in Admixed, Asian, African and European population was listed in Table 2. Out of the eight haplotypes, the frequencies of seven haplotypes were significantly different among the four populations (*P* > 0.01). The frequency of haplotype S-3 is extremely higher in Africa than in other three populations (Fig. 2).

**Table 1:** List of APOL1 gene region haplotypes generated by Tag SNP, which presenting a global frequency higher than 1% considering all populations of the 1000 Genomes Project (Phase 3)

Chr22	Tag SNP	APOL1 position	S-1	S-2	S-3	S-4	S-5	S-6	S-7	S-8
36253756	rs13056427	686	C	T	C	C	C	C	C	C
36256843	rs136147	3773	T	G	G	G	G	G	T	G
36257808	rs10854688	4738	T	C	T	T	C	T	T	C
36258099	rs136150	5029	A	T	A	A	A	A	A	T
36260092	rs136154	7022	T	A	T	T	T	T	T	T
36261550	rs713929	8480	G	A	G	G	G	G	G	G
36263525	rs136165	10455	A	G	A	A	A	A	A	A
36265015	rs28391521	11945	G	A	G	G	G	G	G	G
36265520	rs136175	12450	A	G	A	A	A	A	A	A
36266331	rs9610473	13261	T	T	T	C	T	T	T	T
36266702	rs5750246	13632	A	G	A	A	A	G	G	A
36267202	rs78523	14132	G	A	G	G	G	G	G	G
Global frequency *n* = 2504			0.53607	0.09556	0.08766	0.06525	0.05509	0.02883	0.02758	0.02394

Haplotypes are ordered according to their global frequency

**Table 2:** The most frequent APOL1 haplotypes and their frequencies among the 1000 Genomes Project (Phase 3) in different populations

APOL1 haplotypes	Admixed (*n* = 408)	Africa (*n* = 600)	Asia (*n* = 993)	Europe (*n* = 503)	*P* value
S-1	0.58626	0.55681	0.58872	0.38251	6.50E-14
S-2	0.09184	0.00417	0.10884	0.17979	1.58E-21
S-3	0.07444	0.27567	0.00759	0.02041	1.81E-82
S-4	0.08489	0.0125	0.05652	0.13209	3.26E-14
S-5	0.06474	0.01006	0.03796	0.1339	3.70E-19
S-6	0.02787	0.03067	0.02508	0.04145	0.35
S-7	0.00924	0.00832	0.05031	0.01025	7.76E-09
S-8	0.01121	0.07902	0.00339	0.00501	4.31E-23

### Variants and haplotypes in the upstream regulatory region and 3′ untranslated region of APOL1

There were 79 SNPs in the URR of APOL1 and 92 SNPs in 3’UTR. Total 12 SNPs in URR (Additional file 1: Table S4) and 24 SNPs in 3’UTR (Additional file 1: Table S5) were considered as common variants with MAF ≥ 1%.

**Fig. 3:**
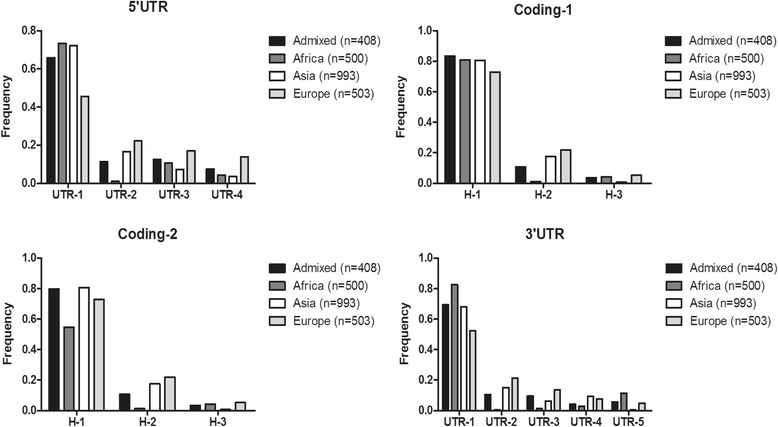
Frequency distribution of different region haplotypes of APOL1 gene in different populations. Coding-1: haplotype analysis not consider the two SNP of G1; Coding-2:haplotype analysis consider the two SNP of G1

Eight SNPs in URR were selected as Tag SNP for haplotype analysis. There were 18 haplotypes emerged, but only 4 haplotypes had a global frequency higher than ≥1% (Table 3). We named the 4 haplotypes as URR-1, URR-2, URR-3, and URR-4 briefly. Considering the global frequency of each haplotype, it was worthy mentioned that the four haplotypes could account for more than 96% of all haplotypes (Table 3). The frequency was significant different among the four populations for each of the 4 haplotypes (Table 4). The frequency of haplotype URR-1 was much lower in European than in other three populations. The haplotype URR-2 was very rare in African populations (Table 4, Fig. 3).

**Table 3:** List of APOL1 upstream regulatory region (URR) haplotypes generated by Tag SNP, which presenting a global frequency higher than 1% considering all populations of the 1000Genomes Project (Phase 3)

Chr22	Tag SNP	APOL1 position	URR-1	URR-2	URR-3	URR-4
36647960	rs4821472	−1157	T	T	T	C
36648093	rs5995271	−1024	G	G	G	T
36648352	rs6000218	−765	A	A	A	C
36648552	rs5756115	−565	A	G	A	A
36648738	rs34318457	−379	C	C	C	T
36649574	rs9610467	458	G	G	A	G
36649802	rs13056427	686	C	T	C	C
36649966	rs6000220	850	C	T	C	C
Global frequency, *n* = 2504			0.66026	0.13199	0.1089	0.0639

Haplotypes are ordered according to their global frequency

**Table 4:** The most frequent APOL1 upstream regulatory region (URR) haplotypes and their frequencies among the 1000 Genomes Project (Phase 3) in different populations

URR haplotypes	Admixed(*n* = 408)	Africa(*n* = 600)	Asia(*n* = 993)	Europe(*n* = 503)	*P* value
URR-1	0.65774	0.73461	0.72127	0.4561	4.80E-27
URR-2	0.11309	0.01062	0.16666	0.22266	4.03E-27
URR-3	0.125	0.1056	0.07228	0.16998	2.38E-07
URR-4	0.07353	0.04312	0.03474	0.13833	1.77E-14

Four SNPs in 3′ UTR region were selected as Tag SNP and produced 9 haplotypes. Five haplotypes reached a global frequency higher than 1% (Table 5). The haplotypes distributed very differently in African than in other three groups: UTR-1 and UTR-5 were much higher, and UTR-2, UTR-3, as well as UTR-4 were much lower in African population (Table 6).

**Table 5:** List of APOL1 3′ untranslated region (3’UTR) haplotypes generated by Tag SNP, which presenting a global frequency higher than 1% considering all populations of the 1000Genomes Project (Phase 3)

Chr22	Tag SNP	APOL1 position	UTR-1	UTR-2	UTR-3	UTR-4	UTR-5
36266331	rs9610473	13261	T	T	C	T	T
36266608	rs66473469	13538	A	A	A	A	C
36266702	rs5750246	13632	A	G	A	G	A
36267202	rs78523	14132	G	A	G	G	G
Global frequency, *n* = 2504			0.68358	0.12093	0.07079	0.06682	0.05027

Haplotypes are ordered according to their global frequency

**Table 6:** The most frequent APOL1 3′ untranslated region (3’UTR) haplotypes and their frequencies among the 1000Genomes Project (Phase 3) in different populations

	Admixed(*n* = 408)	Africa(*n* = 600)	Asia(*n* = 993)	Europe(*n* = 503)	*P* value
UTR-1	0.69348	0.82485	0.68119	0.52248	5.23E-25
UTR-2	0.10433	0.0048	0.15043	0.21333	5.19E-27
UTR-3	0.09513	0.0125	0.06239	0.13618	2.08E-14
UTR-4	0.04153	0.02781	0.09227	0.07494	1.31E-06
UTR-5	0.05685	0.11344	0.00499	0.04771	7.09E-21

### Variants and haplotypes in APOL1 coding region

Only 52 SNPs in APOL1 coding region were recorded in the 1000 Genome database. The MAFs of 11 variant presents at least 1% and 7 of them presented more than 5% (Additional file 1: Table S6). The 11 SNPs were either coding synonymous mutations or missense variants, most of them located in exon 7 (only the first one located in exon 6) (Additional file 1: Figure S3). Seven SNPs were selected as Tag SNPs for haplotype analysis. The three haplotypes with a global frequency higher than 1% were listed in Table 7, named as H-1, H-2 and H-3 haplotype. The frequency of H-2 haplotype was much lower in African population and H-3 was much lower in Asian population (Table 8).

**Table 7:** List of APOL1 coding haplotypes generated by Tag SNP, which presenting a global frequency higher than 1% considering all populations of the 1000 Genomes Project (Phase 3)

Chr22	Tag SNP	APOL1 position	H-1	H-2	H-3
36261694	rs41297245	8624	G	G	A
36265363	rs116136671	12293	A	A	A
36265490	rs136174	12420	A	C	A
36265520	rs136175	12450	A	G	A
36265600	rs136176	12530	A	G	A
36265796	rs136177	12726	A	G	A
36265845	rs16996616	12775	G	G	G
Global frequency, *n* = 2504			0.79577	0.13438	0.02977

Haplotypes are ordered according to their global frequency

**Table 8:** The most frequent APOL1 coding haplotypes and their frequencies among the 1000 Genomes Project (Phase 3) in different populations

	Admixed(*n* = 408)	Africa(*n* = 600)	Asia(*n* = 993)	Europe(*n* = 503)	*P* value
H-1	0.83407	0.80985	0.80661	0.72962	3.33E-04
H-2	0.10634	0.01156	0.17525	0.21869	3.28E-27
H-3	0.03631	0.0425	0.00707	0.05169	9.18E-07

Both MAFs the two SNPs belonging to G1 in Africans were 26%, but both were absent in Asians. In the Hardy–Weinberg equilibrium analysis, HWp < 0.05 indicate that they’re not confirm to Hardy–Weinberg equilibrium and should be discard. Considering the importance of G1 in APOL1 function, we extended our haplotype analysis to include the two SNP. Total 26 haplotypes were observed for the 9 Tag SNPs and 3 haplotypes reached a global frequency higher than 1% (Additional file 1: Table S7). The distribution of the three haplotypes was significantly different among the four populations and was similar to the three haplotypes without G1 variants (Additional file 1: Table S8, Fig. 3). But the frequency of haplotype H-1 decreased in African population when we added the two SNP of G1 for haplotype analysis (Fig. 3).

## Discussions

A majority of rare disease exhibits monogenic pathogenesis and showed the obviously regional differences, population-based genetic studies have identified lots of kidney diseases which had increased genetic risk of developing and progressing. The prevalence of chronic kidney disease (CKD) was increasing worldwide with apparently racial diversity, it indicated that genetic factors played an important role in the development of CKD, looking for CKD susceptibility genes have been becoming the mainstream. Haplotype analysis can help researchers to determine the diseases susceptible genes, and can make a better understanding about the diseases and the patients genotype.

A previous study have shown that several SNPs of APOL1 were significantly associated with ESKD than all previously reported SNPs in MYH9 [39]. In our analysis, we found that the variation of APOL1 gene was common, with up to 613 SNP (1000 Genome Project reported) and 99 of them (16.2%) with MAF > 1%. By describing the data sources and processing of discovery, we found the distribution of these haplotypes were significantly different among different populations, most haplotypes frequency in European present the highest levels than African. The different pattern confirmed the supposition that a stronger signature of balancing selection of APOL1 gene in African.

### APOL1 Coding Region

The 11 selected SNPs in coding region included either synonymous mutations or missense variants, most of them located in exon 7 (only the first one located in exon 6). They jointly participated in the polymorphism of APOL1 four different transcripts and impacted three proteins isoform structure. The limited variants of APOL1 coding region mostly distributed in the PFD, MAD and SRA-interacting domain (Additional file 1: Figure S3) indicated that these SNP has some significant impact for the function of APOL1 protein. The previous study indicated that SP was dispensable for its toxicity, PFD was required but not sufficient for APOL1 mediated toxicity, and integrity of MAD and SRA was critical requirement for the cell injury activity of APOL1 protein [10]. It indicated that those SNPs could contribute a significant effect on the function of APOL1 protein. For example, G1 (rs60910145) played an important role in trypanosome lysis [40] and susceptibility to kidney diseases [26, 27, 41, 42] or schizophrenia [43].

An earlier study reported that about 38% African carried with G1 risk allele [40], in 1000 Genome Project, the MAF of G1 was 26% in African population but absent in Asian and European. Two SNP of G1 didn’t conform to HWp > 0.05, the frequency of G2 also absent in the initial released 1000 Genomes VCF files (Additional file 1: Table S6). Considering the importance of G1 and G2 in APOL1 function [44], we extended our haplotype analysis to include the two G1 SNPs.

The distributions of haplotypes with or without G1 variants were significantly different among the four populations (Fig. 3), G1 two SNP that were previously presented a higher frequency actually pull down the haplotypes frequency when considering all populations pooled together (global frequency). We suspected that the main reason was the two variation sites didn’t conform to the HW equilibrium. The conclusion could safely draw from the two results at different conditions that the influence of two SNPs of APOL1 risk allele G1 in coding region haplotypes seemingly not prominent, it may influence the progress of the relevant diseases independently.

### APOL1 Upstream regulate region and 3’Untranslated region

We also analyzed variants in the URR and 3′ UTR of APOL1. The previous study indicated that there were many regulatory elements in the APOL1 gene upstream, for example, activating protein-1 (AP1) at −1034, sterol regulatory element binding protein (SREBP) sites at −1185, and large number of zinc finger binding sites (MZF1) distribution at APOL1 gene promoter region [14]. In this study, we included the nucleotides between −1200 and +892 to cover all upstream regulatory elements as URR and 1497 nucleotides for 3′ UTR for further analysis.

The URR haplotype URR-1 present similar frequency among Admixed populations, Africans and Asians could indicate it play the same role in the three populations. While an opposite pattern is observed for other three haplotypes, their frequency in European populations is higher than other three populations. In addition, URR-2 is very rare in African populations. The results suggest that URR-1 and URR-2 could play different roles in African and European populations respectively.

The 4 haplotypes of 3’UTR haplotype was significant different among the four populations, UTR-1 appear a highest frequency in Africa populations, this finding suggests a stronger association between UTR-1 and APOL1-related disease in African populations. Haplotype UTR-2 and UTR-3 are absent or rare in populations of African ancestry, and haplotype UTR-5 is absent or rare in Asia and present relative higher frequency in Africa. The result shows the diversity of haplotypes effects on susceptibility of APOL1 gene associated diseases.

Considering we have to construct haplotypes on the basis of the accurate genotypes at huge polymorphic sites, HW equilibrium was always checked using Haploview software, and data deviated strongly from the equilibrium were submitted to retyping or discarded. However, there still some shortcomings in this study: (1) The population composition of Admixed group is relatively complex, it may affect the analysis results, data from Africa, Asia, and Europe are more reliable. (2) A little we know about the effect of intron mutations in APOL1 gene, this study did not analyze the mutations in the intron of APOL1 gene.

## Conclusions

We compared the variants of APOL1 gene among Africa, Europe, Asia and Admixed populations in this study. The results indicated that the distributions of APOL1 gene variants and haplotypes were significantly different among the different populations, in either regulatory or coding regions. It could be helpful for the future genetic study of APOL1 related diseases in different populations.

## Additional file

Additional file 1:
**Table S1.** Size and function of APOL1 exons and introns. **Table S2.** The population and the number of samples in the different regions of people. **Table S3.** List of all SNP (MAF ≥ 1%) found in APOL1 gene region, their genomic positions on chromosome 22 and their allele frequencies presented in 1000 Genomes Project (Phase 3). **Table S4.** List of SNP (MAF ≥ 1%) found in APOL1 upstream regulatory region (URR), their genomic positions on chromosome 22 and their allele frequencies presented in 1000 Genomes Project (Phase 3). **Table S5.** List of SNP (MAF ≥ 1%) found in the APOL1 3′ untranslated region (3’UTR), their genomic positions on chromosome 22 and their allele frequencies presented in 1000 Genomes Project (Phase 3).**Table S6.** List of all SNP found in APOL1 coding region, their genomic positions on chromosome 22 and their allele frequencies presented in 1000 Genomes Project (Phase 3). **Table S7.** List of APOL1 coding haplotypes generated by Tag SNP (consider the two SNP of G1) which presenting a global frequency higher than 1%, considering all populations of the 1000 Genomes Project (Phase 3). **Table S8.** The most frequent APOL1 coding haplotypes and their frequencies (consider the two SNP of G1) among the 1000 Genomes Project (Phase 3) in different populations. **Figure S1.** Linkage disequilibrium plot generated by APOL1 gene SNPs (MAF ≥ 1%). Inter-SNP D’-values are displayed on the plot. **Figure S2.** 12 Tag SNP position in APOL1 gene. **Figure S3.** Spatial distribution of genetic variants at the APOL1 functional domain. (DOCX 531 kb)
