# Supplementary material for: The analysis of APOL1 genetic variation and haplotype diversity provided by 1000 Genomes project
Source: BMC Nephrol. 2017 Aug 11;18:267. doi: 10.1186/s12882-017-0675-6 (PMC5553676; doi:10.1186/s12882-017-0675-6)
Supplement: Additional file 1: — Table S1. Size and function of APOL1 exons and introns. Table S2. The population and the number of samples in the different regions of people. Table S3. List of all SNP (MAF ≥ 1%) found in APOL1 gene region, their genomic positions on chromosome 22 and their allele frequencies presented in 1000 Genomes Project (Phase 3). Table S4. List of SNP (MAF ≥ 1%) found in APOL1 upstream regulatory region (URR), their genomic positions on chromosome 22 and their allele frequencies presented in 1000 Genomes Project (Phase 3). Table S5. List of SNP (MAF ≥ 1%) found in the APOL1 3′ untranslated region (3’UTR), their genomic positions on chromosome 22 and their allele frequencies presented in 1000 Genomes Project (Phase 3).Table S6. List of all SNP found in APOL1 coding region, their genomic positions on chromosome 22 and their allele frequencies presented in 1000 Genomes Project (Phase 3). Table S7. List of APOL1 coding haplotypes generated by Tag SNP (consider the two SNP of G1) which presenting a global frequency higher than 1%, considering all populations of the 1000 Genomes Project (Phase 3). Table S8. The most frequent APOL1 coding haplotypes and their frequencies (consider the two SNP of G1) among the 1000 Genomes Project (Phase 3) in different populations. Figure S1. Linkage disequilibrium plot generated by APOL1 gene SNPs (MAF ≥ 1%). Inter-SNP D’-values are displayed on the plot. Figure S2. 12 Tag SNP position in APOL1 gene. Figure S3. Spatial distribution of genetic variants at the APOL1 functional domain. (DOCX 531 kb) [file 12882_2017_675_MOESM1_ESM.docx]

**Table S1| Size and function of APOL1 exons and introns.**

| **According to**  **NG_023228.1**  **(chrome 22)** | **Size(nt)** | **Function** |
| --- | --- | --- |
| Exon 1 | 149 | 5’UTR |
| Intron 1 | 618 | Spliced out |
| Exon 2 | 154 | 5’UTR/translation initiation |
| Intron2 | 945 | Spliced out |
| Exon3 | 63 | Sig peptide |
| Intron 3 | 2083 | Spliced out |
| Exon 4 | 54 | Sig peptide |
| Intron 4 | 182 | Spliced out |
| Exon 5 | 89 | **NA** |
| Intron5 | 4188 | Spliced out |
| Exon 6 | 127 | Pore forming domain(PFD) |
| Intron 6 | 3428 | Spliced out |
| Exon 7 | 2381 | PD/ Membrane-addressing/  SRA- interacting domain |

**NA: not available**

**Table S2|The population and the number of samples in the different regions of people.**

| **Ethnic group** | **Abbreviation** | **Numbers in APOL1 (Total:2504)** | **Description** |
| --- | --- | --- | --- |
| **Europe(503)** | **GBR** | **91** | **British in England and Scotland** |
|  | **FIN** | **99** | **Finnish in Finland** |
|  | **TSI** | **107** | **Toscani in Italy** |
|  | **IBS** | **107** | **Iberian populations in Spain** |
|  | **CEU** | **99** | **Utah residents with Northern and Western European ancestry** |
| **Asia(993)** | **CHS** | **105** | **Han Southern Chinese, China** |
|  | **CDX** | **93** | **Chinese Dai in Xishuangbanna, China** |
|  | **KHV** | **99** | **Kinh in Ho Chi Minh City, Vietnam** |
|  | **PJL** | **96** | **Punjabi from Lahore,Pakistan** |
|  | **STU** | **102** | **Sri Lankan Tamil from the UK** |
|  | **ITU** | **102** | **Indian Telugu from the UK** |
|  | **BEB** | **86** | **Bengali in Bangladesh** |
|  | **GIH** | **103** | **Gujarati Indian in Houston,TX** |
|  | **CHB** | **103** | **Han Chinese in Bejing, China** |
|  | **JPT** | **104** | **Japanese in Tokyo, Japan** |
| **Admixed(408)** | **PUR** | **104** | **Puerto Rican in Puerto Rico** |
|  | **ASW** | **61** | **African Ancestry in Southwest US** |
|  | **MXL** | **64** | **Mexican Ancestry in Los Angeles, California** |
|  | **CLM** | **94** | **Colombian in Medellin, Colombia** |
|  | **PEL** | **85** | **Peruvian from Lima, Peru** |
| **Africa(600)** | **ACB** | **96** | **African Caribbean in Barbados** |
|  | **GWD** | **113** | **Gambian in Western Division in The Gambia** |
|  | **ESN** | **99** | **Esan in Nigeria** |
|  | **MSL** | **85** | **Mende in Sierra Leone** |
|  | **LWK** | **99** | **Luhya in Webuye, Kenya** |
|  | **YRI** | **108** | **Yoruba in Ibadan, Nigeria** |

**Table S3|List of all SNP (MAF≥1%) found in APOL1 gene region, their genomic positions on chromosome 22 and their allele frequencies presented in 1000 Genomes Project (Phase 3).**

| **Genomic position**  **(Chr22)** | **SNPid** | **APOL1 position** | **Reference MAF** | **Allele 1**  **(reference)** | **Allele1**  **frequency** | **Allele2** | **Allele 2**  **frequency** | **Annotation** |
| --- | --- | --- | --- | --- | --- | --- | --- | --- |
|  |  |  |  |  |  |  |  |  |
| 36253528 | rs9610467 | 458 | A=0.1094/548 | G | 0.8906 | A | 0.1094 | Intronic |
| 36253745 | rs112278014 | 675 | A=0.0212/106 | G | 0.9788 | A | 0.0212 | Intronic |
| 36253756 | rs13056427 | 686 | T=0.1334/668 | C | 0.8666 | T | 0.1334 | Intronic |
| 36253920 | rs6000220 | 850 | T=0.0954/478 | C | 0.9046 | T | 0.0954 | 5' UTR |
| 36254435 | rs13057901 | 1365 | C=0.1012/507 | T | 0.8988 | C | 0.1012 | Intronic |
| 36254910 | rs9610468 | 1840 | A=0.0911/456 | G | 0.9089 | A | 0.0911 | 5' UTR |
| 36255185 | rs28360494 | 1125 | G=0.1164/583 | T | 0.8836 | G | 0.1164 | Intronic |
| 36255267 | rs543736531 | 2197 | T=0.0226/113 | A | 0.9774 | T | 0.0226 | Intronic |
| 36255319 | rs5756118 | 2249 | G=0.4271/2139 | A | 0.5729 | G | 0.4271 | Intronic |
| 36255699 | rs7292413 | 2629 | T=0.0148/74 | C | 0.9852 | T | 0.0148 | Intronic |
| 36256118 | rs7284919 | 3048 | C=0.0994/498 | T | 0.9006 | C | 0.0994 | Intronic |
| 36256698 | rs41302583 | 3628 | G=0.0140/70 | C | 0.9862 | G | 0.0140 | Intronic |
| 36256843 | rs136147 | 3773 | G=0.4109/2058 | G | 0.4109 | T | 0.5891 | Intronic |
| 36256885 | rs136148 | 3815 | C=0.2332/1168 | C | 0.2332 | T | 0.7668 | Intronic |
| 36257229 | rs4820224 | 4159 | A=0.0699/350 | G | 0.9301 | A | 0.0699 | Intronic |
| 36257696 | rs74737052 | 4626 | G=0.1058/530 | C | 0.8942 | G | 0.1058 | Intronic |
| 36257697 | rs136149 | 4627 | G=0.3389/1697 | G | 0.3389 | A | 0.6611 | Intronic |
| 36257706 | rs9610469 | 4636 | G=0.2947/1476 | G | 0.2947 | C | 0.7053 | Intronic |
| 36257808 | rs10854688 | 4738 | C=0.2206/1105 | C | 0.2206 | T | 0.7794 | Intronic |
| 36257884 | rs115869088 | 4814 | T=0.0106/53 | C | 0.9894 | T | 0.0106 | Intronic |
| 36257940 | rs116280906 | 4870 | A=0.0431/216 | C | 0.9569 | A | 0.0431 | Intronic |
| 36258099 | rs136150 | 5029 | T=0.1633/818 | T | 0.1633 | A | 0.8367 | Intronic |
| 36258155 | rs74904227 | 5085 | G=0.0116/58 | C | 0.9884 | AG | 0.0116 | Intronic |
| 36258536 | rs77346339 | 5466 | T=0.0108/54 | C | 0.9892 | T | 0.0108 | Intronic |
| 36258670 | rs2413395 | 5600 | A=0.0248/124 | G | 0.9752 | A | 0.0248 | Intronic |
| 36258966 | rs136151 | 5896 | A=0.1563/783 | A | 0.1564 | G | 0.8437 | Intronic |
| 36258998 | rs115055881 | 5928 | A=0.0128/64 | G | 0.9872 | A | 0.0128 | Intronic |
| 36259215 | rs136152* | 6145 | C=0.0002/1 | C | 0.0002 | T | 0.9998 | Intronic |
| 36259467 | rs136153 | 6397 | C=0.3077/1541 | C | 0.3077 | T | 0.6923 | Intronic |
| 36259617 | rs12106505 | 6547 | T=0.0359/180 | A | 0.9641 | T | 0.0359 | Intronic |
| 36260092 | rs136154 | 7022 | A=0.1368/685 | A | 0.1368 | T | 0.8632 | Intronic |
| 36260179 | rs113661479 | 7109 | A=0.0379/190 | G | 0.9621 | A | 0.0379 | Intronic |
| 36260191 | rs136155 | 7121 | G=0.4579/2293 | G | 0.4579 | A | 0.5421 | Intronic |
| 36260396 | rs136156 | 7326 | C=0.3105/1555 | C | 0.3105 | A | 0.6895 | Intronic |
| 36260397 | rs136157 | 7327 | C=0.3065/1535 | C | 0.3065 | A | 0.6935 | Intronic |
| 36260398 | rs9622362 | 7328 | C=0.1703/853 | A | 0.8297 | C | 0.1703 | Intronic |
| 36260509 | rs9622363 | 7439 | G=0.1683/843 | A | 0.8317 | G | 0.1683 | Intronic |
| 36260607 | rs136158 | 7537 | T=0.3057/1531 | T | 0.3057 | - | 0.6943 | Intronic |
| 36260977 | rs136159 | 7907 | T=0.1366/684 | T | 0.1366 | C | 0.8634 | Intronic |
| 36261073 | rs136160 | 8003 | C=0.3021/1513 | C | 0.3021 | G | 0.6979 | Intronic |
| 36261283 | rs129423 | 8213 | T=0.1362/682 | T | 0.1362 | C | 0.8638 | Intronic |
| 36261386 | rs136161 | 8316 | G=0.4439/2223 | G | 0.4439 | C | 0.5561 | Intronic |
| 36261428 | rs136162 | 8358 | A=0.1314/658 | A | 0.1314 | CG | 0.8686 | Intronic |
| 36261550 | rs713929 | 8480 | A=0.1360/681 | A | 0.1360 | G | 0.8640 | Intronic |
| 36261582 | rs136163 | 8512 | T=0.1362/682 | T | 0.1362 | AG | 0.8638 | Intronic |
| 36261694 | rs41297245 | 8624 | A=0.0302/151 | G | 0.9698 | A | 0.0302 | Missense |
| 36261743 | rs136164 | 8673 | T=0.3349/1677 | T | 0.3349 | C | 0.6651 | Intronic |
| 36262121 | rs55653110 | 9051 | G=0.0797/399 | C | 0.9203 | G | 0.0797 | Intronic |
| 36262124 | rs55647221 | 9054 | -=0.0801/401 | A | 0.9199 | - | 0.0801 | Intronic |
| 36262329 | rs79011803 | 9259 | A=0.0120/60 | G | 0.9880 | A | 0.0120 | Intronic |
| 36262488 | rs713753 | 9418 | C=0.4573/2290 | C | 0.4573 | T | 0.5427 | Intronic |
| 36262758 | rs55690310 | 9688 | C=0.0150/75 | T | 0.9850 | C | 0.0150 | Intronic |
| 36262809 | rs4419330 | 9739 | C=0.0757/379 | T | 0.9243 | C | 0.0757 | Intronic |
| 36262819 | rs4350853 | 9749 | G=0.0393/197 | T | 0.9607 | G | 0.0393 | Intronic |
| 36263142 | rs78469 | 10072 | G=0.4097/2052 | G | 0.4097 | T | 0.5903 | Intronic |
| 36263253 | rs80424 | 10183 | A=0.1346/674 | A | 0.1346 | GC | 0.8654 | Intronic |
| 36263525 | rs136165 | 10455 | G=0.1364/683 | G | 0.1364 | A | 0.8636 | Intronic |
| 36263656 | rs140189796 | 10586 | T=0.0144/72 | C | 0.9856 | T | 0.0144 | Intronic |
| 36264568 | rs136167 | 11498 | A=0.1356/679 | A | 0.1356 | G | 0.8644 | Intronic |
| 36264796 | rs136168 | 11726 | G=0.2774/1389 | G | 0.2774 | A | 0.7226 | Intronic |
| 36264811 | rs557018161 | 11741 | -=0.4529/2268 | T | 0.5471 | - | 0.4529 | Intronic |
| 36264875 | rs136170 | 11805 | C=0.1368/685 | C | 0.1368 | T | 0.8632 | Intronic |
| 36264929 | rs28697951 | 11859 | G=0.1408/705 | A | 0.8592 | G | 0.1408 | Intronic |
| 36265015 | rs28391521 | 11945 | A=0.1300/651 | A | 0.1300 | G | 0.8700 | Intronic |
| 36265103 | rs136169 | 12033 | A=0.1362/682 | A | 0.1362 | G | 0.8638 | Intronic |
| 36265106 | rs28480494 | 12036 | G=0.1390/696 | G | 0.1390 | A | 0.8610 | Intronic |
| 36265284 | rs2239785 | 12214 | G=0.3219/1612 | G | 0.3219 | A | 0.6781 | Missense |
| 36265363 | rs116136671 | 12293 | G=0.0138/69 | A | 0.9862 | G | 0.0138 | Missense |
| 36265490 | rs136174 | 12420 | C=0.1358/680 | C | 0.1358 | AT | 0.8642 | Synonymous |
| 36265520 | rs136175 | 12450 | G=0.1358/680 | G | 0.1358 | A | 0.8642 | Missense |
| 36265600 | rs136176 | 12530 | G=0.1374/688 | G | 0.1374 | A | 0.8626 | Missense |
| 36265796 | rs136177 | 12726 | G=0.1468/735 | G | 0.1468 | A | 0.8532 | Synonymous |
| 36265845 | rs16996616 | 12775 | A=0.0278/139 | G | 0.9722 | A | 0.0278 | Missense |
| 36265860 | rs73885319 | 12790 | G=0.0697/349 | A | 0.9303 | G | 0.0697 | Missense |
| 36265988 | rs60910145 | 12918 | G=0.0695/348 | T | 0.9305 | G | 0.0695 | Missense |
| 36265996 | rs143830837 | 12926-12931 | -=0.0351/176 | ATAATT | 0.9649 | - | 0.0351 | CDS Indel |
| 36266125 | rs116098184 | 13055 | A=0.0132/66 | T | 0.9868 | A | 0.0132 | 3' UTR |
| 36266184 | rs45553833 | 13114 | A=0.0232/116 | G | 0.9768 | A | 0.0232 | 3' UTR |
| 36266325 | rs9610472 | 13255 | G=0.0715/358 | T | 0.9285 | G | 0.0715 | 3' UTR |
| 36266331 | rs9610473 | 13261 | C=0.0715/358 | T | 0.9285 | C | 0.0715 | 3' UTR |
| 36266340 | rs9610474 | 13270 | C=0.0715/358 | T | 0.9285 | C | 0.0715 | 3' UTR |
| 36266608 | rs66473469 | 13538 | C=0.0529/265 | A | 0.9471 | C | 0.0529 | 3' UTR |
| 36266631 | rs9610475 | 13561 | C=0.1282/642 | T | 0.8718 | C | 0.1282 | 3' UTR |
| 36266633 | rs9610476 | 13562 | C=0.1282/642 | T | 0.8718 | C | 0.1282 | 3' UTR |
| 36266643 | rs62233843 | 13573 | C=0.0851/426 | T | 0.9149 | C | 0.0851 | 3' UTR |
| 36266672 | rs140768119 | 13602 | G=0.0477/239 | A | 0.9523 | G | 0.0477 | 3' UTR |
| 36266676 | rs142061305 | 13606 | T=0.0186/93 | C | 0.9814 | T | 0.0186 | 3' UTR |
| 36266677 | rs187937779 | 13607 | A=0.0282/141 | G | 0.9718 | A | 0.0282 | 3' UTR |
| 36266691 | rs151210481 | 13621 | C=0.0218/109 | G | 0.9782 | C | 0.0218 | 3' UTR |
| 36266702 | rs5750246 | 13632 | G=0.1911/957 | G | 0.1911 | A | 0.8089 | 3' UTR |
| 36266712 | rs184571030 | 13642 | C=0.0106/53 | T | 0.9894 | C | 0.0106 | 3' UTR |
| 36266722 | rs189436505 | 13652 | G=0.0224/112 | A | 0.9776 | GT | 0.0224 | 3' UTR |
| 36266778 | rs150349831 | 13708 | A=0.0138/69 | G | 0.9862 | A | 0.0138 | 3' UTR |
| 36266822 | rs138141669 | 13752 | A=0.0136/68 | G | 0.9864 | A | 0.0136 | 3' UTR |
| 36266905 | rs11321251 | 13835 | -=0.1907/955 | A | 0.8093 | - | 0.1907 | 3' UTR |
| 36266912 | rs529136671 | 13842 | C=0.0110/55 | A | 0.9890 | C | 0.0110 | 3' UTR |
| 36267167 | rs58384577 | 14097 | C=0.0685/343 | T | 0.9315 | C | 0.0685 | 3' UTR |
| 36267202 | rs78523 | 14132 | A=0.1252/627 | A | 0.1252 | G | 0.8748 | 3' UTR |
| 36267287 | rs1142542 | 14217 | A=0.0873/437 | G | 0.9127 | A | 0.0873 | 3' UTR |
| 36267490 | rs3075462 | 14420-14421 | -=0.1360/681 | TG | 0.8640 | - | 0.1360 | 3' UTR |

**The underline bases: reference alter allele in 1000 Genome Project**

**Table S4|List of SNP (MAF ≥ 1%) found in APOL1 upstream regulatory region (URR), their genomic positions on chromosome 22 and their allele frequencies presented in 1000 Genomes Project (Phase 3).**

| **Genomic position(Chr22)** | **SNPid** | **APOL1**  **position** | **Allele 1**  **(reference)** | **Allele 1**  **frequency** | **Allele 2** | **Allele 2**  **frequency** |
| --- | --- | --- | --- | --- | --- | --- |
|  |  |  |  |  |  |  |
| 36251914 | rs4821472 | -1157 | T | 0.8666 | C | 0.1334 |
| 36252001 | rs541608939 | -1070 | C | 0.9892 | T | 0.0108 |
| 36252047 | rs5995271 | -1024 | G | 0.8319 | T | 0.1681 |
| 36252306 | rs6000218 | -765 | A | 0.9042 | C | 0.0958 |
| 36252506 | rs5756115 | -565 | A | 0.8660 | G | 0.1340 |
| 36252573 | rs118162445 | -498 | C | 0.9878 | G | 0.0122 |
| 36252692 | rs34318457 | -379 | C | 0.9357 | T | 0.0643 |
| 36253528 | rs9610467 | 458 | G | 0.8906 | A | 0.1094 |
| 36253745 | rs112278014 | 675 | G | 0.9788 | A | 0.0212 |
| 36253756 | rs13056427 | 686 | C | 0.8666 | T | 0.1334 |
| 36253920 | rs6000220 | 850 | C | 0.8091 | T | 0.1909 |
| 36253958 | rs139494181 | 888 | C | 0.9900 | T | 0.0100 |

**Table S5|List of SNP (MAF ≥ 1%) found in the APOL1 3’ untranslated region (3’UTR), their genomic positions on chromosome 22 and their allele frequencies presented in 1000 Genomes Project (Phase 3).**

| **Genomic position**  **(Chr22)** | **SNPid** | **APOL1**  **position** | **EXON**  **position** | **Allele 1**  **(reference)** | **Allele1**  **frequency** | **Allele 2** | **Allele 2**  **frequency** |
| --- | --- | --- | --- | --- | --- | --- | --- |
| 36266125 | rs116098184 | 13055 | EXON 7 | T | 0.986821 | A | 0.013179 |
| 36266184 | rs45553833 | 13114 | EXON 7 | G | 0.9768 | A | 0.0232 |
| 36266325 | rs9610472 | 13255 | EXON 7 | T | 0.9285 | G | 0.0715 |
| 36266331 | rs9610473 | 13261 | EXON 7 | T | 0.9285 | C | 0.0715 |
| 36266340 | rs9610474 | 13270 | EXON 7 | T | 0.9285 | C | 0.0715 |
| 36266608 | rs66473469 | 13538 | EXON 7 | A | 0.9471 | C | 0.0529 |
| 36266631 | rs9610475 | 13561 | EXON 7 | T | 0.8718 | C | 0.1282 |
| 36266633 | rs9610476 | 13562 | EXON 7 | T | 0.8718 | C | 0.1282 |
| 36266643 | rs62233843 | 13573 | EXON 7 | T | 0.9149 | C | 0.0851 |
| 36266672 | rs140768119 | 13602 | EXON 7 | A | 0.9523 | G | 0.0477 |
| 36266676 | rs142061305 | 13606 | EXON 7 | C | 0.9814 | T | 0.0186 |
| 36266677 | rs187937779 | 13607 | EXON 7 | G | 0.9718 | A | 0.0282 |
| 36266691 | rs151210481 | 13621 | EXON 7 | G | 0.9782 | C | 0.0218 |
| 36266702 | rs5750246 | 13632 | EXON 7 | G | 0.1911 | A | 0.8089 |
| 36266712 | rs184571030 | 13642 | EXON 7 | T | 0.9894 | C | 0.0106 |
| 36266722 | rs189436505 | 13652 | EXON 7 | A | 0.9776 | GT | 0.0224 |
| 36266778 | rs150349831 | 13708 | EXON 7 | G | 0.9862 | A | 0.0138 |
| 36266822 | rs138141669 | 13752 | EXON 7 | G | 0.9864 | A | 0.0136 |
| 36266905 | rs11321251 | 13835 | EXON 7 | A | 0.8093 | - | 0.1907 |
| 36266912 | rs529136671 | 13842 | EXON 7 | A | 0.9890 | C | 0.0110 |
| 36267167 | rs58384577 | 14097 | EXON 7 | T | 0.9315 | C | 0.0685 |
| 36267202 | rs78523 | 14132 | EXON 7 | A | 0.1252 | G | 0.8748 |
| 36267287 | rs1142542 | 14217 | EXON 7 | G | 0.9127 | A | 0.0873 |
| 36267490 | rs3075462 | 14420-14421 | EXON 7 | TG | 0.8640 | - | 0.1360 |

**The underline bases: reference alter allele in 1000 Genome Project.**

**Table S6| List of all SNP found in APOL1 coding region, their genomic positions on chromosome 22 and their allele frequencies presented in 1000 Genomes Project (Phase 3).**

| **Genomic position**  **(Chr22)** | **SNPid** | **APOL1 position** | **EXON**  **position** | **Allele 1**  **(reference)** | **Allele 1**  **frequency** | **Allele2** | **Allele 2**  **frequency** | **Annotation** |
| --- | --- | --- | --- | --- | --- | --- | --- | --- |
| 36261694 | rs41297245 | 8624 | EXON 6 | G | 0.969848 | A | 0.030152 | missense |
| 36265284 | rs2239785 | 12214 | EXON 7 | G | 0.321885 | A | 0.678115 | missense |
| 36265363 | rs116136671 | 12293 | EXON 7 | A | 0.986222 | G | 0.013778 | missense |
| 36265490 | rs136174 | 12420 | EXON 7 | C | 0.135783 | AT | 0.864217 | Synonymous |
| 36265520 | rs136175 | 12450 | EXON 7 | G | 0.135783 | A | 0.864217 | missense |
| 36265600 | rs136176 | 12530 | EXON 7 | G | 0.13738 | A | 0.86262 | missense |
| 36265796 | rs136177 | 12726 | EXON 7 | G | 0.146765 | A | 0.853235 | synonymous |
| 36265845 | rs16996616 | 12775 | EXON 7 | G | 0.972244 | A | 0.027756 | missense |
| 36265860 | **rs73885319** | 12790 | EXON 7 | A | 0.930312 | G | 0.069688 | missense |
| 36265988 | **rs60910145** | 12918 | EXON 7 | T | 0.930511 | G | 0.069489 | missense |
| 36265996 | rs143830837 | 12926-12931 | EXON 7 | ATAATT | 0.964856 | - | 0.035144 | indel |
| 36266000 | **rs71785313** | 12930-12935 | EXON 7 | TTATAA | NA |  | NA | indel |

**Bold SNP: APOL1 risk variants G1 (rs73885319 and rs60910145) and G2 (rs71785313). The underline bases: reference alter allele in 1000 Genome Project.**

**Table S7|List of APOL1 coding haplotypes generated by Tag SNP (consider the two SNP of G1) which presenting a global frequency higher than 1%, considering all populations of the 1000 Genomes** **Project (Phase 3).**

| **Chr22** | **Tag SNP** | **APOL1 position** | **H-1** | **H-2** | **H-3** |
| --- | --- | --- | --- | --- | --- |
| 36261694 | rs41297245 | 8624 | G | G | A |
| 36265363 | rs116136671 | 12293 | A | A | A |
| 36265490 | rs136174 | 12420 | A | C | A |
| 36265520 | rs136175 | 12450 | A | G | A |
| 36265600 | rs136176 | 12530 | A | G | A |
| 36265796 | rs136177 | 12726 | A | G | A |
| 36265845 | rs16996616 | 12775 | G | G | G |
| 36265860 | rs73885319 | 12790 | A | A | A |
| 36265988 | rs60910145 | 12918 | T | T | T |
| **Global frequency, n=2504** | | | 0.73255 | 0.13438 | 0.02851 |

**Haplotypes are ordered according to their global frequency.**

**Table S8|The most frequent APOL1 coding haplotypes and their frequencies (consider the two SNP of G1) among the 1000 Genomes Project (Phase 3) in different populations.**

|  | **Admixed (n=408)** | **Africa**  **(n=600)** | **Asia**  **(n=993)** | **Europe**  **(n=503)** | ***P* value** |
| --- | --- | --- | --- | --- | --- |
| **H-1** | 0.79767 | 0.54627 | 0.80661 | 0.72962 | **3.61E-30** |
| **H-2** | 0.10623 | 0.01167 | 0.17525 | 0.21869 | 3.28E-27 |
| **H-3** | 0.0341 | 0.0425 | 0.00707 | 0.05169 | 9.30E-07 |


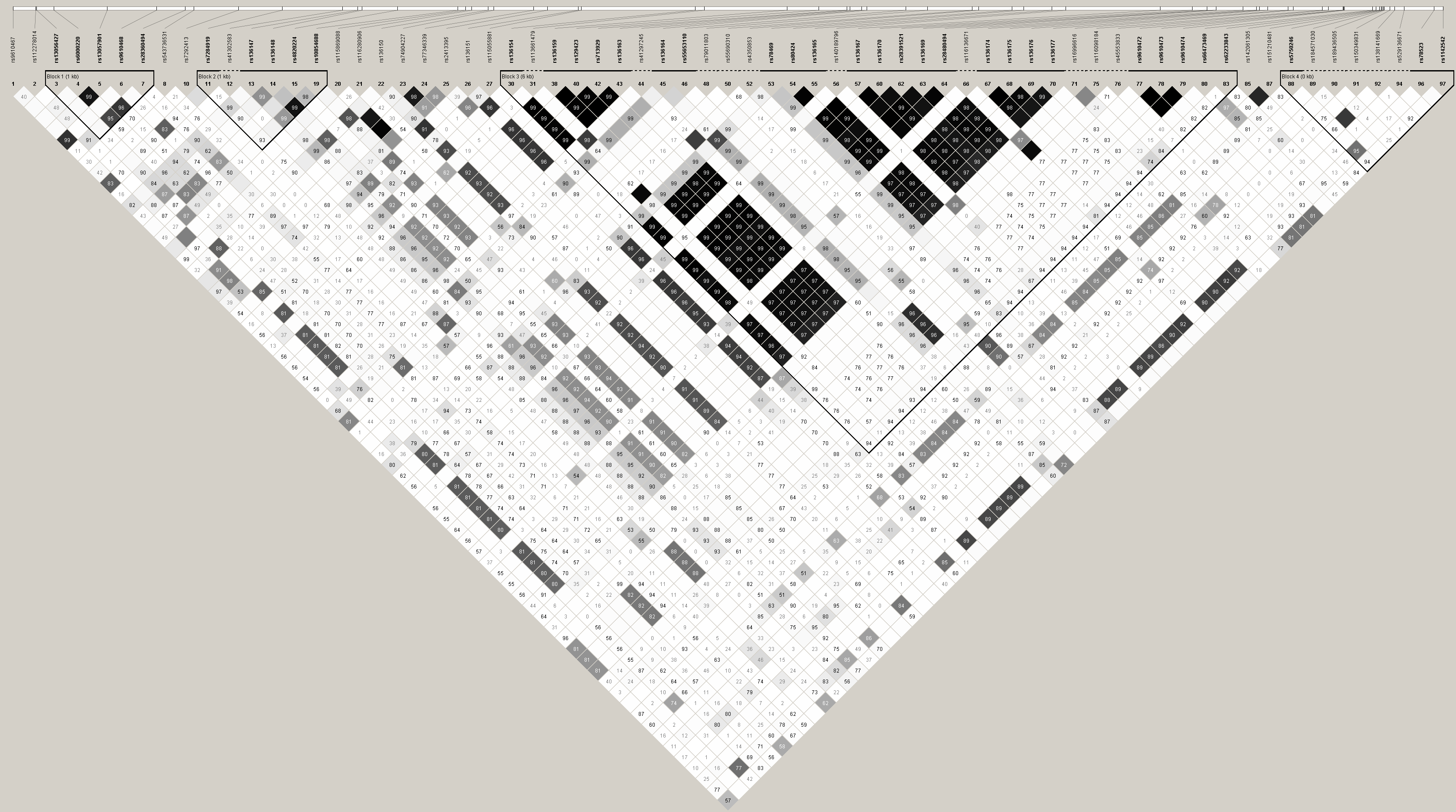


**Figure S1|Linkage disequilibrium plot generated by APOL1 gene SNPs (MAF ≥ 1%). Inter-SNP D’-values are displayed on the plot.**

**Darker grey represents regions of high pairwise r^2^ and white represents regions of low pairwise r^2^; The numbers in the boxes are the pairwise r^2^ values.**

**
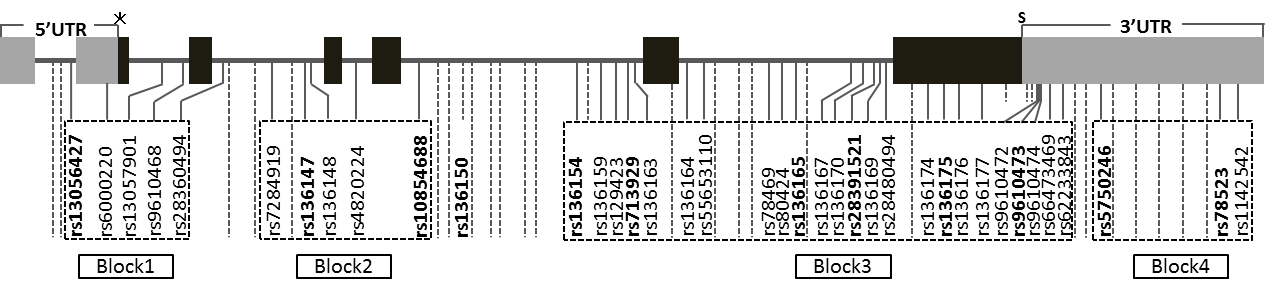
**

**Figure S2|12 Tag SNP position in APOL1 gene.**

**Black box: haplotype block region; bold SNP be selected as tag SNP.**

**Figure S3|Spatial distribution of genetic variants at the APOL1 functional domain.**
